# Infant EEG theta modulation predicts childhood intelligence

**DOI:** 10.1038/s41598-020-67687-y

**Published:** 2020-07-08

**Authors:** E. J. H. Jones, A. Goodwin, E. Orekhova, T. Charman, G. Dawson, S. J. Webb, M. H. Johnson

**Affiliations:** 1 Centre for Brain and Cognitive Development, Birkbeck, University of London, Malet Street, London, WC1E 7HX UK; 2 Institute of Psychiatry, King’s College London, 16 De Crespigny Park, Camberwell, London, SE5 8AF UK; 3 MedTech West and the Institute of Neuroscience and Physiology, Sahlgrenska Academ, The University of Gothenburg, Kungsgatan 12, SE 411 19 Gothenburg, Sweden; 4 Duke Centre for Autism and Brain Development, Department of Psychiatry and Behavioral Sciences, Duke University, 4584 White Zone, Duke South, Durham, 27705 NC USA; 5 Center On Human Development and Disability, University of Washington, 1701 NE Columbia Rd, Seattle, WA 98195 USA; 6 Department of Psychiatry & Behavioral Science, University of Washington, 2815 Eastlake Ave E, Seattle, WA 98102 USA; 7 Center On Child Behavior and Development, Seattle Children’s Research Institute, 2001 Eighth Ave, Suite 400, Seattle, WA 98121 USA; 8 MEG Centre, Moscow State University of Psychology and Education, 123290 Moscow, Russia

**Keywords:** Intelligence, Human behaviour

## Abstract

Intellectual functioning is a critical determinant of economic and personal productivity. Identifying early neural predictors of cognitive function in infancy will allow us to map the neurodevelopmental pathways that underpin individual differences in intellect. Here, in three different cohorts we investigate the association between a putative neurophysiological indicator of information encoding (change in frontal theta during a novel video) in infancy and later general cognitive outcome. In a discovery cohort of 12-month-old typically developing infants, we recorded EEG during presentation of dynamic movies of people and objects. Frontal theta power (3–6 Hz) significantly increased during the course of viewing each video. Critically, increase in frontal theta during viewing of a video was associated with a differential response to repetition of that specific video, confirming relation to learning. Further, individual differences in the magnitude of change in frontal theta power were related to concurrent nonverbal cognitive level. We then sought to extend this association in two independent samples enriched for variation in cognitive outcome due to the inclusion of infants at familial risk for autism. We observed similar patterns of theta EEG change at 12 months, and found a predictive relation to verbal and nonverbal cognitive skills measured at 2, 3 and 7 years of age. For the subset of high-risk infants later diagnosed with autism, infant theta EEG explained over 80% of the variance in nonverbal skills at age 3 years. We suggest that EEG theta change in infancy is an excellent candidate predictive biomarker that could yield substantial insight into the mechanisms that underlie individual differences in childhood intelligence, particularly in high risk populations.

## Introduction

By school start, most children have an extensive vocabulary, recognise colours and shapes, and can remember and match pictures and objects. Such abilities are acquired through interaction between the child and his or her environment. Individual differences in childhood cognitive function predict longer term outcomes (e.g.^1–4^, particularly at the extremes^5,6^ and in neurodevelopmental conditions such as autism spectrum disorder (ASD)^7–9^. Cognitive delays are commonly associated with most developmental risk factors, including premature birth^10^, low birth weight^11^, under-nutrition^12^, toxin exposure^13^, and genetic conditions^14^. Identifying the brain mechanisms that underpin individual differences in information encoding in infancy could lead to substantial theoretical insights into developmental origins of core cognitive skills, and improve our ability to rapidly identify children at heightened risk for poor cognitive development.

In the late twentieth century, the search for infant predictors of later cognitive functioning focused on looking time (e.g.^15,16^). Measures such as habituation or attention to novelty show moderate correlations (of around 0.4) with later cognitive function^17–19^ that are partially mediated through processing speed and memory skills^20,21^. However, such associations are restricted to early infancy and may only be useful in a relatively brief developmental window^19^. Further, the degree of variance captured by early looking time measures is relatively small (4–10%; 22). Progress requires looking beyond behaviour to the new availability of infant-friendly methods to directly measure brain activity. Electroencephalography (EEG) provides a non-invasive method that measures the oscillatory states involved in memory and learning. EEG is sensitive to the dynamics of neural processing, and thus the effects of information encoding on brain systems. Advances in EEG technology allow brain activity to be recorded during natural behaviour, raising the possibility of real-time access to the brain rhythms underpinning information encoding.

In the present study, we focused on a theoretically motivated analysis of dynamic changes in theta power elicited by ecologically valid stimuli. A substantial body of evidence links increases in theta power to processes important in information encoding (such as attention, learning and memory), particularly in the low-theta range (e.g.^23–25^. Locking of neuron firing to theta oscillations is tightly coupled to better memory^26^, and stimuli presented during particular phases of theta oscillations produce greater synaptic plasticity^27^. Theta oscillations measured from intra-cranial hippocampal recordings are in phase synchrony with similar oscillations recorded over the scalp, supporting the possibility of information transfer^25^. Indeed, increases in frontal theta power have been linked to the encoding of new information^28^, as well as novelty detection and memory maintenance^29,30^. Gamma-band activity (associated with a range of cognitive processes) is also modulated by theta power^31,32^. Selective^33^ and spatial attention^34^ have also been linked to theta oscillations and their relation to gamma activity. Further, in infancy, frontal theta power measured during object manipulation correlates with subsequent recognition of that object^35^. Infants show increases in theta power during events likely to be associated with learning or memory, including anticipation of a face reappearing during peekaboo^36^, during active exploration of new toys or people^37^, when expectations are violated^38^, and while infants are waiting to retrieve a hidden object^39^. Taken together, this literature provides preliminary evidence that changes in theta power (particularly over frontal cortex) are associated with information encoding in infancy, but to date there are no reports that individual differences in these responses are related to later cognitive outcome.

### Present study

In the present study, we test whether dynamic modulation of theta power in response to ecologically valid stimuli is related to individual differences in general intelligence later in development in populations enriched for varied cognitive outcome. We introduce a method of measuring theta modulation suitable for application in naturalistic contexts that simulate a child’s natural learning environment. We focus on *change* in theta within each video because this metric has been previously linked to neural plasticity during internal control of attention, learning and memory formation^40^. To do this, we leveraged data from three cohorts of young infants. Cohort 1 was a large cross-sectional study of typically developing 12-month-old infants. Within this dataset, we establish that viewing dynamic semi-naturalistic videos produced increases in theta power, and critically that change in theta within the first presentation of a specific video predicts subsequent responses to the same stimulus (confirming an association to learning and memory). In a subset of these infants, we then tested whether changes in theta power relate to concurrent cognitive skills. Cohorts 2 and 3 were independent studies of 12-month-old infants at low and high familial risk for autism followed longitudinally. In these cohorts, we examine whether dynamic modulation of frontal theta power predicts later intelligence at 2, 3 and 7 years in a population at heightened risk for cognitive delay. For all cohorts, parents provided informed consent for study participation. All data is available from the authors on request.

## Dynamic modulation of theta rhythms and concurrent cognitive skill

Cohort 1 was a cross-sectional study of typically developing 12-month-old infants. From a larger dataset, we focused on EEG data recorded while infants watched dynamic videos of women talking and toys moving^41^. Each video set was repeated twice, separated by around 10 min of other stimuli (static faces and toys). Within this dataset, we first established that our stimuli produce increases in frontal theta over time; that this change predicts specific responses to that video on repetition (consistent with learning and memory); and asked whether individual differences in frontal theta response are associated with concurrent measures of cognitive skill.

### Methods

#### Participants

Participants were 106 12-month-old (51 female) typically developing full-term infants (see SI1.1 for inclusion/exclusion). Infants were only included if they had sufficient artifact-free data for each analysis (see SI1.2 for details). Parents and their infants were recruited using a University Infant Participant Pool. Table SI1 shows demographic and descriptive data for the full sample. Of note, we have previously reported overall condition and region effects on EEG-theta in these infants^41^, but we did not examine dynamic change within a video. The study was ethically reviewed and approved by the University of Washington Institutional Review Board and conducted in accordance with all relevant guidelines and regulations; all parents provided informed consent.

#### EEG

 EEG was recorded in a shielded room from 128-channel Geodesic sensor nets; recorded online with reference to the vertex; digitization at 500 Hz; amplification at 1,000 × and band-pass filtering at 0.1 to 100 Hz. Children were presented with two movie sets of 1-min duration repeated twice during the session; order of presentation was counterbalanced. Movies were (a) Social: 4 nursery rhymes with gestures told by two women; (b) Non-Social: 5 vignettes of child-appropriate dynamic toys (e.g. balls dropping down a chute). Following gold-standard procedures described fully in SI1.2.1^42^, EEG data were segmented into continuous 1-s segments with no overlap and epochs of visual attention to the screen selected; artifacts (e.g. motion, poor signal) were identified; bad channels were interpolated (Netstation 4.1); and the remaining data was re-referenced to the average. We then extracted natural-logged power in the theta (3–6 Hz) frequency band using fast-fourier transform. Of note, definitions of frontal theta in infancy vary between studies within the range of a lower bound of 2–3 and an upper bound of 5–6; we selected 3–6 Hz because it represents the modal definition of theta in previous work^43^. Our primary index was percent change in theta power between the first and second halves of the first presentation of each video (frontal theta during second half-frontal theta during first half/frontal theta during first half), because the second repetition would be contaminated by familiarity. When examining associations with cognitive skill, we addressed the question of specificity by additionally examining %change in posterior theta power (spatial specificity), %change in scalp high alpha power (band specificity) and %change in the proportion of clean attended segments, a proxy for a behavioral measure of focused attention^44^ that provides an indication of whether similar effects could be obtained from measuring behavior alone.

#### Cognitive testing

The Mullen Scales of Early Learning^45^ is a cognitive assessment for infants from birth to 5 years. The measure was administered by a trained experimenter (EJ) for approximately half the sample. Scores fall into five scales measuring fine motor and visual reception skills (nonverbal cognition), receptive and expressive language skills (verbal cognition) and gross motor skills. Raw scores were converted into t scores using age-appropriate norms. Of note, all statistical tests are two-tailed.

#### Data and code availability

Data and Matlab code will be made available on request to the corresponding author, subject to conformance with the ethical approvals with which it was collected. This process is managed through the BASIS network policies and procedures (www.basisnetwork.org.uk).

### Results

**Figure 1:**
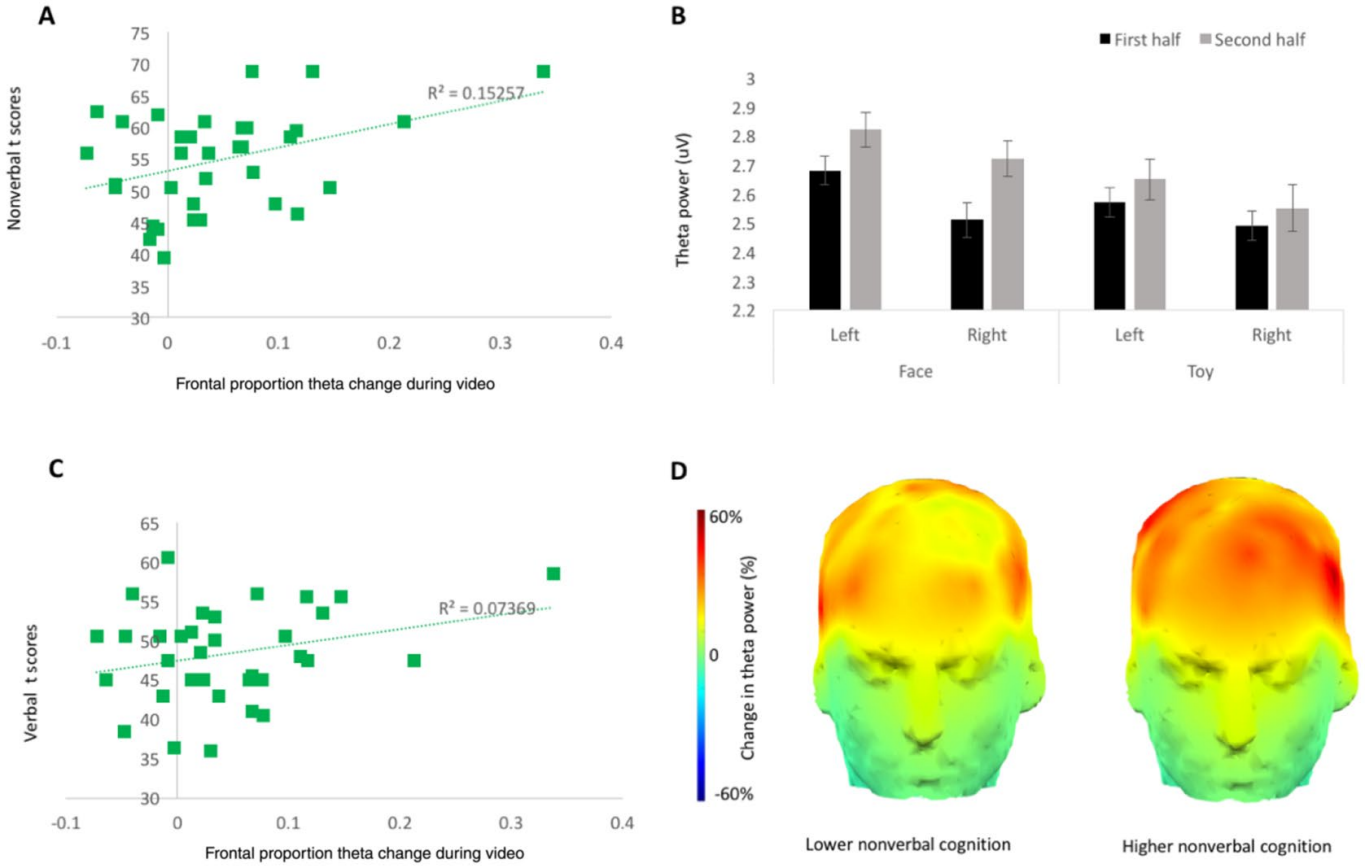
Relation between modulation of theta power and cognitive skills in Cohort 1. **A** Greater frontal theta change within a video significantly related to higher nonverbal scores at 12 months, but not verbal scores; removal of the most extreme frontal theta change score rendered the association with nonverbal skills marginal (r(33) = 0.26, p = 0.1). (**C**). **B** Theta power varied by condition, region, and half of video. Error bars are ± 1 SE. **D** Illustration of the topography of change in theta power over the course of the videos in children with low and high cognitive ability (based on a median split of nonverbal standard scores at 12 months).

We first established that our semi-naturalistic videos produced an increase in frontal theta between the first and second halves of the first presentation of each video (Fig. 1B; n = 36, 21 female). In an ANOVA on theta power by condition (social, non-social), video segment (first half, second half) and region (left, right) we found expected main effects of condition (social > non-social: *F*(1,35) = 11.14*, p* = 0.002, η^2^ = 0.24), and region (left > right (*F*(1,35) = 7.05*, p* = 0.012, η^2^ = 0.17); and confirmed that theta power increased between the first and second halves of the video (main effect of half: *F*(1,35) = 7.02*, p* = 0.012, η^2^ = 0.17). The magnitude of the half effect did not interact with condition or region (*p*s > 0.3). Thus, for subsequent analyses we collapsed across condition and region to maximise the sample size. This allowed us to include additional infants who had sufficient data in only one of the two conditions.

To establish whether frontal theta power relates to cognitive skills, we used a subset of the infants (n = 34, 18 female) who had theta data and the verbal and nonverbal subdomains of the Mullen Scales of Early Learning (only collected on half our sample due to resource limitations). In this subsample, frontal theta also increased during the video presentation (*F*(1,33) = 10.87, *p* = 0.002). We then computed Pearson correlations between change in frontal theta and verbal and nonverbal cognitive level. Greater frontal theta change was related to higher nonverbal cognitive level (*r*(34) = 0.39, *p* = 0.022; Fig. 1A) but not significantly to verbal cognitive level (*r*(34) = 0.27, *p* = 0.12; Fig. 1C). Results were not confounded by gender or data quantity (SI1.3). Proportion change in clean attended segments between the first and second halves of the video was not greater than zero (proportion change M = 0.068, SD = 0.43, one-sample t test against zero *t*(33) = 0.93, *p* = 0.36) and was not associated with nonverbal cognitive level (*r*(34) = − 0.06, *p* = 0.63) or % frontal theta change (*r*(34) = 0.089, *p* = 0.62) indicating similar effects could not be observed purely from related behavioral measures.

To test specificity to the theta band, we computed power in the upper alpha band (8 to 9 Hz, to avoid contamination from theta activity). There were no significant relations between frontal alpha change and cognitive skills at 12 months (nonverbal *r*(34) = − 0.22*, p* = 0.22; verbal *r*(34) = − 0.21*, p* = 0.23). Of note the trends were negative, in line with the proposed inverse relation between theta and alpha power, and between alpha suppression and increased semantic encoding^46,47^. Given this sign difference, the correlations between theta and alpha power and cognitive skills were significantly different (Steiger method; verbal: Z = 2.35, p = 0.009; nonverbal: Z = 1.83, p = 0.033). Further, to establish that our results were specific to frontal scalp regions, we examined relations between cognitive skills and change in theta power over posterior cortex. This revealed no significant association (nonverbal *r*(34) = 0.16*, p* = 0.38; verbal *r*(34) = 0.20*, p* = 0.27).

**Figure 2:**
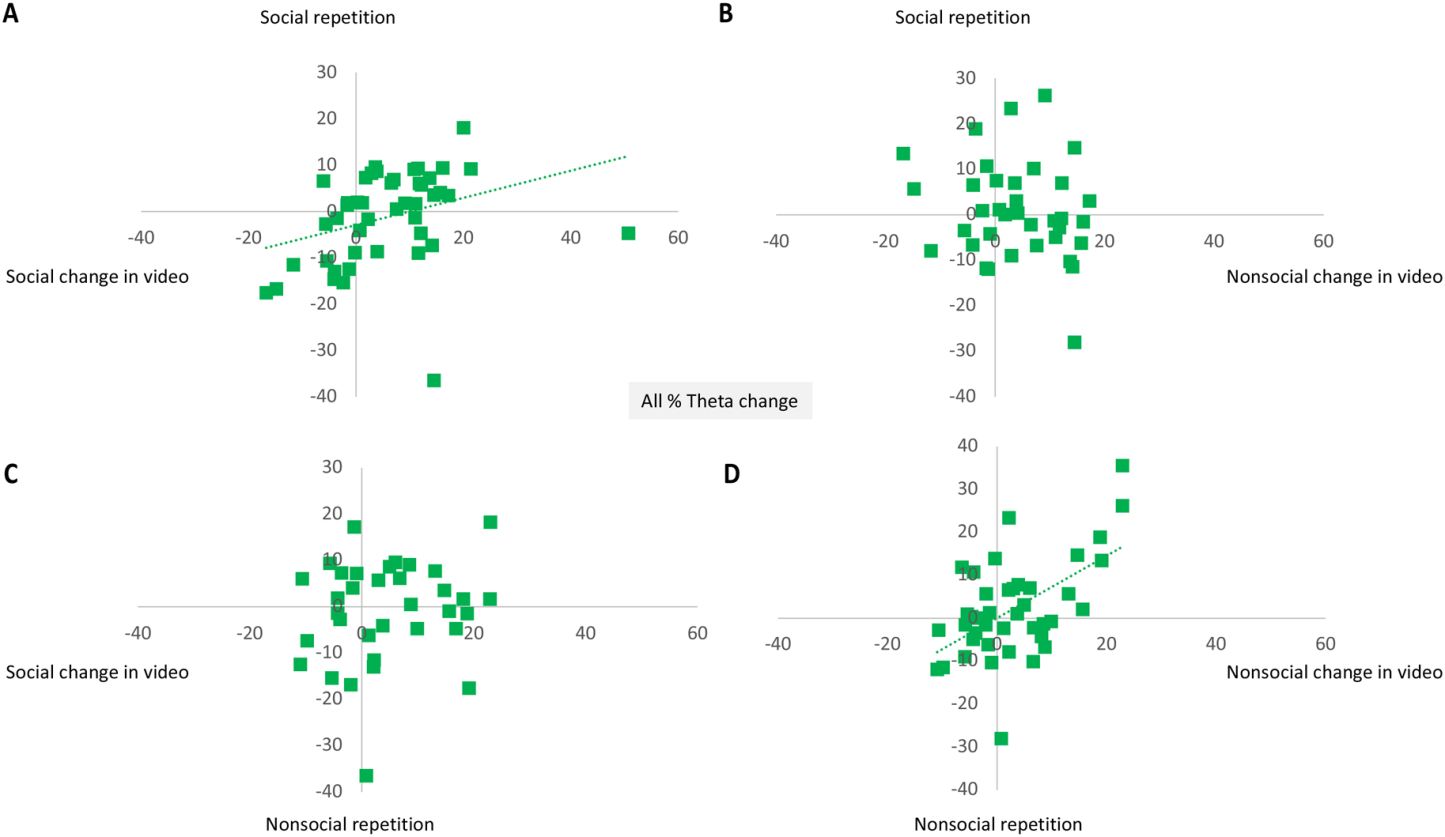
Relation between % frontal theta change within a video (x axes), and % frontal theta change on subsequent repetition of that video (y axes; Cohort 1). Greater percent frontal theta change within the social video predicts change in frontal theta between the two video repetitions (**A**) but not change in frontal theta between non-social repetitions (**C**); greater percent theta change within the nonsocial video predicts change in frontal theta between the two non-social video repetitions (**B**) but not change in frontal theta between social repetitions (**D**).

To establish whether change in frontal theta power during the first presentation of a video reflects learning, we examined correlations with change in frontal theta power between the first and second presentations of that specific video in all infants with data available (partialling out total frontal theta power during the first half of the first social/non-social video). A greater change in frontal theta during the first social video predicted a greater change in theta between the first and second repetitions of the social video (*r*(43) = 0.40, *p* = 0.007; Fig. 2A); but not the non-social video (*r*(32) = − 0.08, *p* = 0.65; Fig. 2C). Likewise, a greater change in frontal theta during the first nonsocial video predicted a greater change in frontal theta between the first and second repetitions of the non-social video (*r*(38) = 0.56, *p* < 0.001; Fig. 2D); but did not predict greater change in frontal theta between the first and second repetitions of the social video (*r*(30) = 0.12, *p* = 0.52; Fig. 2B). Thus, this provides support for the hypothesis that change in frontal theta power reflects a learning mechanism specific to one type of video.

## Longitudinal prediction of cognitive skill

Using data from two independent cohorts assessed with a comparable EEG protocol, we built on the findings from Cohort 1 to ask whether dynamic change in frontal theta power at 12 months could predict cognitive skills later in development in cohorts enriched for varied cognitive development. Specifically, we used data from cohorts of infants at high familial risk for ASD because they have an older sibling with a diagnosis; such infants are at risk for a range of outcomes including lower verbal and nonverbal skill^48^. Cohort 2 was 43 infants at high familial risk for ASD, with EEG data collected at 12 months and developmental and cognitive data at 24 months (see SI2.1 and 2.2). The study was ethically reviewed and approved by the University of Washington Institutional Review Board and conducted in accordance with all relevant guidelines and regulations; all parents provided informed consent. Cohort 3 was an independent sample of 54 high risk and 50 low risk infants with EEG recorded at 14 months and cognitive and developmental skills measured at 36 months and 7 years (see SI3.1 and 3.2). The study was ethically reviewed and approved by the London Central NHS Research Ethics Committee; all parents provided informed consent. All infants at high risk for autism had an older sibling with a community clinical diagnosis of ASD; all low risk infants had an older sibling with typical development. Cognitive assessments used the Mullen Scales of Early Learning. Assessment of autism outcome was made based on ADOS, ADI, and the clinical judgement of highly experienced clinical teams. For Cohort 2, the EEG paradigm was identical to Cohort 1. For Cohort 3, videos were remade to suit the UK context (SI3.2.2). Processing pipelines were substantially similar to Cohort 1, though since data were initially processed at a different Centre, minor details of the processing pipeline were different (SI3.2.2). Given the findings in Cohort 1, we collapsed across condition and region and simply tested the relation between change in frontal theta and later cognitive skills. We chose to use existing data for comparability to other reports^49^; if our effects are robust, they should generalise across common variability in EEG processing pipelines. Full details of both samples, children included in final analyses and associated methods can be found in the SI.

### Results

**Figure 3:**
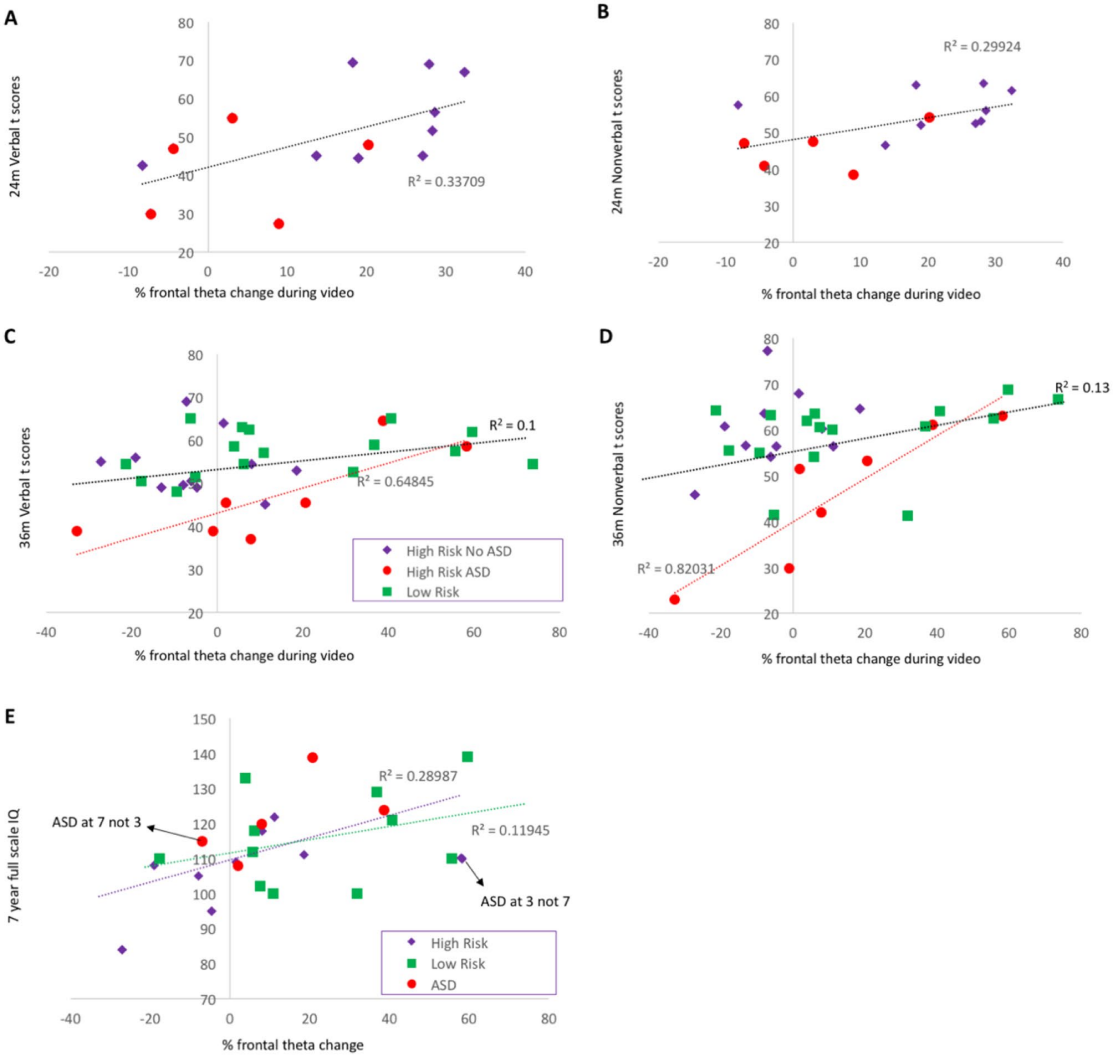
Relation between frontal theta change at 12 months and verbal (**A**) and nonverbal skills (**B**) at 24 months in infants at high risk for ASD (Cohort 2); Relation between frontal theta change at 12 months and verbal (**C**) and nonverbal (**D**) ability at 36 months and IQ at age 7 (**E**) in infants at low and high risk for ASD (Cohort 3); for **C** and **D** the black regression line illustrates the relation within the group of all infants, and the red line illustrates the relation within the group with ASD only.

In Cohort 2, frontal theta change in n = 14 12-month-old high risk infants with EEG and cognitive data correlated with greater nonverbal skills at 24 months (*r*(14) = 0.55, *p* = 0.043; Fig. 3B; with 12-month cognitive skill covaried *r*(11) = 0.51, *p* = 0.08). This was also true for verbal skills (*r*(14) = 0.58, *p* = 0.029; Fig. 3A; controlling for 12-month nonverbal skills *r*(11) = 0.69, *p* = 0.009); this was not confounded by data quantity (SI2.3). Infants later diagnosed with ASD are depicted in Fig. 3, but our power was too low to examine this group separately. Similar associations were observed for percent change in posterior theta power (nonverbal: *r*(14) = 0.57, *p* = 0.034; verbal: *r*(14) = 0.70, *p* = 0.006) but not for scalp alpha power (*r*(14) = 0.28, *p* = 0.33; verbal: *r*(14) = 0.31, *p* = 0.28), indicating a degree of specificity to the frequency band but less specificity to the scalp region. Neither 24 m outcome associated with proportion change in clean attended segments, indicating that measures of behaviour alone are unlikely to capture the same effect (nonverbal: *r*(14) = − 0.21, *p* = 0.48; verbal: *r*(14) = − 0.18, *p* = 0.55).

In Cohort 3, we had sufficient power to examine whether the relation between infant theta and later cognition varied by risk or outcome status. We used an ANCOVA to examine the relation between percent change in frontal theta and nonverbal cognitive skills at 36 months by outcome group (Low Risk n = 16, High Risk No ASD n = 11, High Risk ASD n = 7), the latest time-point with cognitive data available for the whole sample (see SI3.3.2 for outlier analysis). Greater change in frontal theta related to higher nonverbal cognitive skills at 36 months across groups (*F*(1,34) = 14.18, *p* = 0.001, η^2^ = 0.34; Fig. 3D; controlling for 12-month nonverbal skills *F*(1,33) = 13.02, *p* = 0.001, η^2^ = 0.33). There was a significant interaction with group (*F*(2,34) = 5.46, *p* = 0.01, η^2^ = 0.28, controlling for 12-month nonverbal skills *F*(1,33) = 4.97, *p* = 0.015, η^2^ = 0.28). Figure 3D shows that this reflected stronger relations within the ASD group (*r*(7) = 0.91, *p* = 0.005); this was not confounded by data quantity (SI3.3.1). Across the cohort, 36 m nonverbal cognitive skills did not associate with proportion change in posterior theta power (*F*(1,34) = 0.47, *p* = 0.50, η^2^ = 0.017), associated weakly with scalp alpha power (*F*(1,34) = 3.57, *p* = 0.069, η^2^ = 0.11), and did not associate with proportion change in clean attended segments (*F*(1,34) = 1.25, *p* = 0.27, η^2^ = 0.043), indicating a degree of specificity.

Greater change in frontal theta also marginally related to higher verbal skills (*F*(1,34) = 2.84, *p* = 0.1, η^2^ = 0.09, controlling for 12-month nonverbal skills *F*(1,33) = 2.32, *p* = 0.14, η^2^ = 0.08), but this varied by group (*F*(1,34) = 3.77, *p* = 0.036, η^2^ = 0.21; controlling for 12-month nonverbal skill *F*(1,33) = 3.95, *p* = 0.032, η^2^ = 0.23). Relations were only significant within the ASD group (*r*(7) = 0.81, *p* = 0.029; Fig. 3C). Across the cohort, 36 m verbal cognitive skills did not associate with proportion change in posterior theta power (*F*(1,34) = 1.32, *p* = 0.26, η^2^ = 0.045), associated weakly with scalp alpha power (*F*(1,34) = 3.64, *p* = 0.067, η^2^ = 0.12), and did not associate with proportion change in clean attended segments (*F*(1,34) = 0.38, *p* = 0.54, η^2^ = 0.014), indicating a degree of specificity.

Children in Cohort 3 were also followed up at age 7 years (see SI4), at which point their IQ was measured using the Weschler Abbreviated Intelligence Scale (WASI). For children with sufficient data (n = 14 high risk and n = 11 low risk) we used an ANCOVA with risk group as a factor to test whether relations between infant frontal theta change and later WASI total score varied by risk status (group sizes were too small to examine by ASD outcome). There was a significant overall relation between 14-month frontal theta and 7-year IQ (*F*(1,25) = 5.27, *p* = 0.03, η^2^ = 0.2); this did not significantly vary by risk group (*F*(1,25) = 0.34, *p* = 0.57, η^2^ = 0.016 ; r values shown in Fig. 3E). WASI total score at 7 did not associate with proportion change in posterior theta power (*F*(1,25) = 0.43, *p* = 0.52, η^2^ = 0.019), or scalp alpha power (*F*(1,25) = 0.65, *p* = 0.43, η^2^ = 0.029) and did not associate with proportion change in clean attended segments (*F*(1,25) = 1.16, *p* = 0.29, η^2^ = 0.052), indicating specificity.

## General discussion

Frontal theta power has been previously linked to information encoding in young infants^35^. Here, we show that viewing dynamic semi-naturalistic videos elicits an increase in theta power that predicts subsequent change in EEG response when that specific video is repeated. In three independent samples, we show that individual differences in the percentage change in frontal theta during dynamic videos at 12 months is associated with variation in verbal and nonverbal cognitive skills concurrently at age one, and longitudinally at 2, 3 and 7 years. Taken together, our results show that task-dependent modulation of frontal theta power during the first year of life is strongly associated with individual differences in concurrent and later cognitive development.

We interpret our results as consistent with a process that is the outcome of the coordinated functioning of learning, memory and attention systems. The semi-naturalistic design of the study means we cannot precisely elucidate the cognitive mechanisms that underlie the observed changes in frontal theta power. However, we believe that the frontal theta changes observed in response to the first novel video are most likely to closely track the encoding of information, rather than its retrieval. The videos used in the present study were novel to the infant, and the five individual vignettes that made up the videos were each different. Thus, the change in frontal theta between the first and second half of the video does not reflect recognition of repeated content. Interestingly, we have recently replicated the observation that change in frontal theta during an unfamiliar video predicts later cognitive ability in infancy in a pre-registered study that used a series of cartoon vignettes with which infants were highly unlikely to be previously familiar^50^. Thus, although in the present study infants may have had some pre-existing familiarity with the nursery rhymes shown or the toys displayed, this is unlikely to explain the present results. Further, the present design was limited by the lack of a specific set of memory probes designed to determine the precise content of what infants might be learning. However, we were able to show that change in theta within the first video predicted change in theta from the first to the second presentation of that same video (but not a different video), suggestive of an encoding-related signal. Indeed, a wide body of literature indicates that 12-month-old infants can learn information similar to that presented in our semi-naturalistic videos (nursery rhymes with actions and object functions) through passive observation^51,52^, and indeed learn one to two novel behaviors a day through watching and imitating^53^. Thus, observations from the present study are consistent with the possibility that change in frontal theta during the first presentation of a novel video can index encoding-relevant processes.

Previous work also indicates an important role for frontal theta in learning/encoding. Frontal theta may guide learning in conditions of uncertainty, such as when stimuli are novel^54^. Indeed, increased cross-frequency coupling between frontal theta and parietal gamma oscillations has been linked to better subsequent memory in adults^55^; as has coherence between frontal and occipital theta oscillations^56^. While identification of neural generators is not feasible in our data set, frontal theta power during heart-rate defined epochs of sustained attention at 10- and 12- months has been sourced to generators in the orbital frontal, temporal pole, and ventral temporal areas^57^; stimuli presented during heart-rate defined epochs of sustained attention are subsequently better remembered^58^. Direct bidirectional connections between the medial prefrontal cortex and the hippocampus exist in both rodents and humans that facilitate encoding and retrieval of memories from contextual details^59^ and memory traces are formed in frontal cortex during an initial experience^60^. The dynamic changes in theta power observed in the present study may reflect the coordinated action of attention and memory systems in service of encoding the semi-naturalistic events presented. This may represent an important canalization process through which multiple individual cognitive challenges (e.g. poor attention, poor memory, altered processing of prediction) could result in impoverished learning and thus compromise later outcomes. Further, the observed relation between change in theta power at 12 months and cognitive function at 2, 3 and 7 were present when concurrent cognitive ability was covaried. This is consistent with the possibility that modulation of theta power by naturalistic stimuli is a causal mechanism that leads to optimal learning and better later cognitive development. Identifying processes that may mediate the effects of a range of risk factors on cognitive development is critical to finding generalizable mechanisms to target for early intervention, an approach that could also provide a test of our causal hypothesis.

We propose that our findings on the dynamic modulation of EEG theta open up new avenues for the discovery of biomarkers that predict cognitive development from infancy. Our demonstration of promise at 12 months is valuable, because this is a common age for developmental screening in many countries (enabling advanced neuroimaging techniques to be potentially useful as second-stage screeners). This goal is critically important. Significant cognitive delays are seen in up to 40% of children born extremely prematurely^10^; 30% of children who experience malnutrition^12^; and up to 50% of children with ASD, although these are less apparent in our familial risk sample^61^. However, we currently have a very poor ability to predict which infants will experience a poor prognosis. Predicting later trajectories would allow us to focus interventions more effectively. Further, we need outcome measures in infancy that can determine whether our interventions are affecting the cognitive systems that will shape long term outcomes. Current behavioural assessments of infant cognitive development are often affected by environmental opportunity^62^, and child willingness to engage with the testing process^63^. Behavioural cognitive assessments measure *what* a child has learned, and thus are very hard to make culturally ‘fair’. Moving from a focus on domain-general measures of the skills an infant has already learned towards measuring the brain systems that shape their capacity to learn over time will be a critical step for translational research.

We propose that focusing on dynamic modulation of EEG theta could deliver substantial advances in our ability to identify children at risk for a poor cognitive prognosis. We need to optimise our protocols to enable measurement of EEG theta change in a larger proportion of infants tested. However, our data are highly promising—although the group size is small, infant theta power predicted 82% of the variance in verbal skills at age 3 years within children with ASD. Because we utilised data from existing studies, there were small differences in the pipelines between Cohorts (particularly 2 and 3). We view this as a strength of the study, in that similar results were obtained despite minor methodological differences. Indeed, our recent pre-registered replication of these effects^50^ that again uses a set of existing data initially pre-processed for another purpose confirms the robustness of our effects. Although we selected the modal definition of the theta band (3–6 Hz) used in previous studies^43^, optimisation may also include determining the precise frequency band in which modulation occurs through using paradigms designed to elicit longer segments of data that afford greater frequency resolution. Further, the specificity of the observed results to particular scalp regions or frequency bands is important to further probe. Although frontal theta was most consistently associated with cognitive skill, in some analyses additional relations were observed with alpha power or with posterior theta. The degree to which this may reflect spillover from the target frequency band or volume conduction should be examined in future work with protocols optimised for source analysis. However, it was notable that a highly comparable proxy measure for behavioral attention (percent change in the number of clean attended segments) was not associated with cognitive function in any analysis. This highlights the importance of more direct measures of brain function, although future work should examine whether the use of a greater number of video angles would allow the use of a more nuanced behavioural coding scheme that might be more sensitive than the proxy measure used here. Coupling measures of infant theta power with early predictors of ASD outcome^64^ would be important. Further, relations between infant theta power and cognitive skills were observed across three independent cohorts. Intriguingly, relations with the high-risk group and particularly children with autism were stronger than in the low risk group, in line with other evidence that the stability of IQ over time is higher in high risk populations^5–9^. Combining EEG theta with other measures that may be sensitive to categorical autism outcome^49,64^ may further improve predictive validity. Infants will vary on a series of dimensions, and developing a measure battery that is sensitive to the most important of those may ultimately tell us which infants are at relatively greater risk for a particular set of cognitive outcomes^65^.

In our search for biomarkers, we should be looking for the mechanisms that shape learning from the environment in infancy; determine association with later functioning; and finally testing whether an intervention that targets the purported mechanism can improve later cognition. Indeed, in a recent study, we showed that indices of theta power during the same videos appeared to be altered by parent-mediated intervention in infants at familial risk for ASD^66^, and interventions that improve verbal IQ in children with ASD also strengthen theta responses to faces^46^. Our emerging understanding of the mechanisms shaping theta oscillations in the brain^67^, could also create the possibility for developing and testing medications for children at high risk.

## Conclusions

We show that dynamic modulation of frontal theta power in response to novel semi-naturalistic stimuli predicts cognitive skills at age 1, 2, 3 and 7 years in low and high-risk populations. Our work yields new insight into the neural substrates underlying increasing top-down control of learning and memory in infancy, and generates new directions for the stratification of cognitive outcomes in at-risk populations.

## Supplementary information

Supplementary information
