# Supplementary material for: Infant EEG theta modulation predicts childhood intelligence
Source: Sci Rep. 2020 Jul 8;10:11232. doi: 10.1038/s41598-020-67687-y (PMC7343785; doi:10.1038/s41598-020-67687-y)
Supplement: Supplementary file 1 — Supplementary information [file 41598_2020_67687_MOESM1_ESM.pdf]

Supplementary Information

Infant EEG theta modulation predicts childhood intelligence

Authors and affiliations

[1]Jones, E.J.H.*, [2]Goodwin, A., [3]Orekhova, E., [2]Charman, T., [4]Dawson, G., [5,6,7]Webb, S.J., & [1]Johnson, M.H.

*Corresponding Author: Emily J.H. Jones, Centre for Brain and Cognitive Development, Birkbeck, University of London, Malet Street, London, WC1E 7HX. +44 (0) 2076316323; e.jones@bbk.ac.uk, orcid.org/0000-0001-5747-9540.

[1]Centre for Brain and Cognitive Development, Birkbeck, University of London, Malet Street, London, WC1E 7HX.

[2]Institute of Psychiatry, King's College London, 16 De Crespigny Park, Camberwell, London, SE5 8AF.

[3]Gillberg Neuropsychiatry Centre, University of Gothenburg, Kungsgatan 12, SE 411 19, Gothenburg, Sweden.

[4]Department of Psychiatry and Behavioral Sciences, Duke University, 4584 White Zone, Duke South, Durham NC 27710.

[5]Center on Human Development and Disability, University of Washington, 1701 NE Columbia Rd, Seattle WA, 98195.

[6]Department of Psychiatry & Behavioral Science, University of Washington, 2815 Eastlake Ave E, Seattle WA, 98102.

[7]Center on Child Behavior and Development, Seattle Children's Research Institute, 2001 Eighth Ave, Suite 400, Seattle WA, 98121.

1. Cohort 1

1.1. Further participant information: Infants were reported to be developing typically by their parents. Additional exclusion criteria assessed through a parental screening form included: physical signs (e.g., dysmorphic features) of known genetic syndromes, serious medical or neurological conditions (e.g., encephalitis, concussion, seizure disorder, diabetes, congenital heart disease), neurocutaneous markings, or sensory impairments such as vision or hearing loss; serious motor impairment; birth weight < 2000 grams and/or gestational age < 37 weeks, history of intraventricular hemorrhage, exposure to neurotoxins (including alcohol, drugs), and maternal gestational diabetes. In addition, variables that may impact family functioning (e.g., serious parental substance abuse, bipolar disorder, or psychosis) were exclusion criteria.

1.2.1 Methods for Artifact Detection: Continuous EEG data was segmented into consecutive one-second segments with no overlap. Artifact detection of EEG data was accomplished with both automatic artifact-detection software (NetStation 4.3) and through hand-editing (EJ). Segments were rejected if the signal amplitude exceeded 250 μV, or if electro-ocular, movement or muscular artifact occurred. Channels with noisy data were interpolated by an algorithm incorporated within NetStation 4.3 (segments were excluded from analysis if more than 20% of channels were subject to interpolation, or if there were more than 5 interpolated channels within a scalp region). Data was then re-referenced to the average reference, and the resulting segmented data was imported into Matlab.

Within Matlab (using in-house algorithms), segments were detrended and subjected to an FFT, producing power spectra for electrodes grouped within a priori regions approximately equating to F3/F4 (frontal left: 24,28,29,25,21,20; frontal right: 3,4,124,123,119,118). For each segment, data from electrodes with a power value of more than 3 standard deviations

from the mean of the remaining electrodes in a topographical group in the frequency bands of interest were dropped. Power values were then averaged across artifact-free segments and electrodes within topographical groups and within each half of each video repetition; natural logs were calculated to reduce skew. Finally, logged power values were averaged across the theta (3 to 6Hz) frequency range. Participants were only included in analyses if they provided at least 10 artifact-free trials per video half/condition (e.g. first half of the social video set); this represents a minimum cut-off of approximately a third of the duration of each video (20 seconds out of the total video duration of 60 seconds). We have previously published group-level data on averaged theta power (but not change in theta power or relation to IQ) from this cohort[1].

1.2.2 Methods - Final sample: For the first set of analyses (theta change by condition, region and half) we included all infants who had sufficient EEG data for each half of both the social and non-social video. Of the original sample of 106 typically developing infants, 36 had sufficient data for this analysis. For examination of relation to cognitive skills, we collapsed across condition and region, yielding greater availability of EEG data because infants with data from one condition could be included. 67 infants had sufficient EEG data for this analysis (an inclusion rate of approximately 63%), of whom 33 infants did not have data available on the Mullen Scales of Early Learning. This is because we originally only administered the Mullen to approximately 50% of this large sample, due to time constraints. Thus, the final included sample for the relation to cognitive skill was 34 typically developing infants.

Total sample (female)<br><br>Number with EEG and cognitive data	106 (51)<br><br>34 (18)
Age (days) *M* (*SD*)<br><br>Range	378.2 (12.0)<br><br>361-448
First Child	58%
Primary Caregiver (Mother | Employed)	95% | 95%
Participant Race<br><br>(Caucasian, Asian, More than one race)	79%, 4%, 17%
Primary: College Education<br><br>(None, Some, Graduate)	1%, 9%, 89%
Family Income<br><br>(<$35k, $35-75k, >$75)	6%, 32%, 65%,

***Table S1**: Participant demographics for the full sample of infants.*

<< INSERT TABLE 1 ABOUT HERE>>>

		Trial numbers
Social	1st half	21.2 (5.44)
	2nd half	19.2 (5.08)
Nonsocial	1st half	21.3 (5.37)

	2nd half	19.6 (4.52)

***Table S2**: Average trials per period. Values are mean (standard deviation).*

1.3.1 Results- Trial Number: Table S2 shows the average number of trials available per segment for children included in analysis. An ANOVA on trial numbers shows that there were significantly fewer trials for the second half of the video ($F(1,35) = 6.9, p = 0.013, \eta^2=0.17$). Thus, we repeated the analysis of theta change including the difference in trial availability between the first and second half of the video (collapsed by condition) as a covariate; as in the main analysis this showed a significant effect of condition ($F(1,34) = 9.34, p = 0.004, \eta^2 = 0.22$) and half ($F(1,34) = 4.75, p = 0.036, \eta^2 = 0.12$). We then covaried the same trial number metric from correlations with cognition. As in the main analysis greater frontal theta change was related to higher nonverbal cognitive level ($r(31) = 0.42, p = 0.016$) but not significantly to verbal cognitive level ($r(31) = 0.31, p = 0.083$).

1.3.2 Results- Sex: We also examined whether any of the main analyses differed between male and female infants. For the analysis of theta power by condition, region and half, the main effect of half remained ($F(1,34) = 6.58, p = 0.015, \eta^2 = 0.16$). The only significant effect of gender was an interaction between condition, half, region and gender ($F(1,34) = 5.33, p = 0.027, \eta^2 = 0.14$). Follow-up analyses suggested that increase in theta power between the first and second half of the video was bigger for boys over the right vs left hemisphere for non-social videos ($F(1,14) = 6.14, p = 0.027, \eta^2 = 0.31$). However, since this was not predicted we did not interpret this pattern further. The relation between nonverbal scores and frontal theta change during the video remained significant ($F(2,33) = 5.29, p = 0.028, \eta^2 = 0.15$) and did not vary by gender (interaction $F(2,33) = 0.51, p = 0.48, \eta^2 = 0.016$).

2. Cohort 2

2.1 Further participant information: Infants were from a larger sample of 43 high-risk infants followed longitudinally as part of the Early Connections Study at the University of Washington[2]. Low-risk controls in this study did not receive cognitive assessments at 24 months. Inclusion criteria for high-risk infant siblings included age (≤ 6 months), presence of autism in a full biological older sibling, and anticipated residence in the region (within 1.5 hours driving distance from the University) for the next 2 years. To confirm the diagnosis of ASD in an older sibling, the Autism Diagnostic Interview-Revised (ADI-R) was administered by phone and medical records were collected to confirm the diagnosis was based on DSM-IV criteria from a psychologist or physician. Additional exclusion criteria included: physical signs (e.g., dysmorphic features) of known genetic syndromes, serious medical or neurological conditions (e.g., encephalitis, concussion, seizure disorder, diabetes, congenital heart disease), neurocutaneous markings, or sensory impairments such as vision or hearing loss; serious motor impairment; birth weight < 2000 grams and/or gestational age < 37 weeks, history of intraventricular hemorrhage, exposure to neurotoxins (including alcohol, drugs), and maternal gestational diabetes. In addition, variables that may impact family functioning (e.g., serious parental substance abuse, bipolar disorder, or psychosis) were exclusion criteria.

2.2.1 Methods - Autism Assessment: At 24 months, infants in the HR group were administered the ADOS module 1 (Lord et al., 2000). The ADOS is a play-based observation scale administered by a trained research reliable examiner. Infants are engaged in a semi-structured play with developmentally appropriate activities designed to elicit early social-communicative behaviours, language and communication, play and stereotyped/restricted behaviours or interests. The researcher also completed the Autism Diagnostic Interview-Revised (Lord, Rutter & Le Couteur, 1994) with the child’s primary caregiver. Further, a

clinical diagnosis was given as defined in the DSM-IV (American Psychiatric Association, 1994) by the consensus judgment of a certified clinical assessor and a licensed clinical psychologist, based on all available information obtained through the ADOS, ADI-R, cognitive testing, and any other experiences with the infants. Clinicians who were involved in diagnostic judgments are highly experienced in assessing ASD in toddlers of this age. Based on this information, infants were classified as having "Autistic Disorder", "Pervasive Developmental Disorder- Not Otherwise Specified" (collapsed into ASD) or "no diagnosis". Clinicians judged their confidence in the classification as "Very confident", "Somewhat confident", or "Not confident".

2.2.2 Methods – Artifact detection: Collection and processing of infant EEG data were identical to those described in Experiment 1. Trained clinicians administered the Mullen Scales of Early Learning at 24 months. We have previously published group-level data on averaged theta power (but not change in theta power or relation to IQ) from this cohort[3].

2.2.3 Methods - Final sample: Of the original sample of 43 high-risk infants, 28 did not provide sufficient EEG data at 12 months and one did not receive a 24-month cognitive assessment. Thus, the final included sample was 14 infants with an older sibling with a clinical diagnosis of ASD (confirmed with the ADI-R) who provided both sufficient data in an EEG assessment at 12 months and a cognitive assessment at 24 months. For visualization (but not analysis, given the small sample size), infants within the HR group were further divided based on their diagnostic outcome at 24 months. Of the included group of 14 HR infants, infants in the HR-ASD (n=5) group all met DSM IV criteria for ASD at 24 months. Where clinicians had judged that they were "Not confident" in this judgment, infants were

additionally required to meet cut-off on the ADOS for ASD (n=1). Infants in the HR-ASD-No ASD group were judged to have "no diagnosis" on DSM-IV criteria (n=9).

2.2.4 Methods - Analysis strategy: We focused on percent change in frontal theta between the first and second half of the first presented video set (collapsed across condition), and its relation to nonverbal cognitive skills at 24 months (the oldest age to which children were followed in this sample). We required infants to have at least 12 segments in the first and second half of the social/non-social video. We computed simple correlations between change in theta power and later verbal/nonverbal skills.

	High Risk (n=14)
N trials 1st half	27.4 (9.2)
N trials 2nd half	29.2 (9.9)
12 month Nonverbal t-score	58.9 (6.3)
24 month verbal t-score	49.9 (12.9)
24 month nonverbal t-score	52.4 (7.7)

Table S3*: Profile of infants included in analyses.*

2.3 Results - Effects of trial number: In a repeated measures ANOVA the number of segments included in the first and second halves of the videos did not significantly differ ($F(1,23) = .72$, $p = 0.40$).

3. Cohort 3

3.1 Further participant information: Participants came from a larger sample of 104 children taking part in a prospective longitudinal study of infants at high- and low- familial risk for autism (hereafter, HR and LR). Siblings completed research visits at 7 and 14 months of age, around their second and third birthdays, and were invited to return at age 6-8 years.
At enrolment, each HR infant (n = 54) had an older sibling (in 4 cases, a half-sibling) with a community clinical ASD diagnosis, confirmed using information from the Development and Well-Being Assessment (DAWBA; Goodman et al., 2000) and the Social Communication Questionnaire (SCQ; Rutter et al., 2003) by expert clinicians on our team (TC, PB)[1]. Parent-reported family medical histories were examined for significant conditions in the proband or extended family members (e.g., Fragile X syndrome, tuberous sclerosis) with no such exclusions deemed necessary. LR controls (n = 50) were full-term infants recruited from a volunteer database. Medical history review confirmed lack of ASD within first-degree relatives. At enrolment, all LR infants had at least one older sibling. The SCQ was used to confirm absence of ASD in these older siblings, with no child scoring above instrument cut-off ($\geq$ 15; n = 1 missing data).

3.2.1 Methods - Autism Assessment: Four clinical researchers (KH, SC, GP, TC) reviewed information across the research visits at 2 years (including ADOS-G, Mullen Scales of Early Learning (MSEL; Mullen 1995) and Vineland assessments) and 3 years (including MSEL, Vineland, ADOS-G and ADI-R) and assigned clinical diagnoses of ASD according to ICD-10 (World Health Organization, 1993). For the LR control children, in the absence of a full

[1] 5 DAWBA and 5 SCQ missing

developmental history (no ADI-R was administered) no formal clinical diagnoses were considered but none had a community clinical ASD diagnosis.

3.2.2 Methods - EEG Acquisition and artifact detection: Since data was collected at a separate Centre and initially processed for a separate purpose [4], methods for Cohort 3 were slightly different from Cohort 1 and 2. We chose not to reanalyze this dataset to facilitate later integration with other EEG analyses of this dataset, and also because if our measures are to provide a robust biomarker, they should generalise across subtle differences in processing pipelines. Briefly, infants sat on their parents' laps at a 60 cm distance from a 40 x 29 cm CRT monitor. Continuous EEG was sampled while participants watched three types of video stimuli, each lasting for 30-40 sec: (1) a woman singing nursery rhymes or playing peek-a-boo ('social' video); (2) brightly colored toys moving and producing sounds ('non-social' video); and (3) the same sounding toys manipulated by a human hand ('non-social' video). Infants' behaviour (looking, gross body/head/arm movements, crying) and distracting events (e.g. parent's speech, sucking of pacifier, etc.) were coded off-line. The infant was rated as watching the video when she/he looked at the screen, did not move and was not distressed. All participants included in the analysis looked at the screen for at least 85% of time and did so without motion or negative affect for more than 65% of the time.

EEG was recorded using a 128-electrode Hydrocel Geodesic Sensor Net (EGI, Eugene, OR) with respect to the vertex and sampled at 500 Hz. Twelve ridge electrodes most often contaminated by artifacts were excluded from analysis resulting in 116-electrode layout. Data preprocessing and analysis was performed using FieldTrip (http://fieldtrip.fcdonders.nl/) as well as in-house software. The behavioural coding results were synchronised with EEG and the periods when the baby was not looking at the screen, moved, or cried, as well as the periods of interference were excluded from analysis. EEG was visually inspected for artifacts. The bad

channels were interpolated and data were segmented to 1-s segments with 50% overlap and re-referenced to the grand average of 116 channels. Full details on data pre-processing are also reported elsewhere [4,5]. Fast Fourier transform (FFT) was used to calculate theta (3-5 Hz) spectral power in the first and second halves of the videos. Frontal theta power values were obtained by averaging the power in a selection of frontal channels (3, 4, 5, 9, 10, 11, 12, 14, 15, 16, 18, 19, 21, 22, 23). Note that electrode numbering for this cap is slightly different to the Geodesic Sensor Nets (2.1) used in Cohorts 1 and 2, but electrode regions were substantially overlapping. The power values were then log-transformed to reduce skew. EEG was divided into the first and second halves of the first presentation of a particular stimulus condition (20 second segments); percent change was computed as above. Participants were included if they provided at least 10 segments of artifact-free EEG for each of the time intervals. We have previously published group-level data on averaged theta power (but not change in theta power or relation to IQ) from this cohort [4].

3.2.3 Methods - Final sample: Of the original sample of 54 high-risk infants and 50 low-risk infants, 35 high risk and 34 low risk infants did not provide sufficient EEG data at 12 months. The higher attrition rate in this sample is likely because the videos were shorter than those used for Cohorts 1 and 2. One infant (with ASD outcome) was considered an outlier; see below for analyses including this child. Thus, the final included sample was 16 low risk infants and 18 high risk infants, of whom 7 were considered to have ASD at 36 months.

3.2.4 Methods - Analysis strategy: Following the results of Cohort 1 and 2 and to avoid multiple comparisons, we focused on percent change in frontal theta between the first and second half of the video (collapsed across condition), and its relation to nonverbal cognitive skills at 36 months (the oldest age to which children were followed in this sample). This was

analysed in an ANCOVA in which nonverbal cognitive skills were entered as the dependent variable; with outcome group (HR-ASD, HR-no ASD, LR), theta change, and the interaction between theta change and group as predictors. We then examined whether relations remained if nonverbal cognitive skill at 12 months were covaried, and whether relations were also observed for verbal skills, following the findings of Experiment 2.

	Low Risk (n=16)	*High Risk no ASD (n=11)*	*High Risk ASD (n=7)*
N trials 1st half	23.6 (6.9)	22.0 (7.4)	21.3 (8.99)
N trials 2nd half	18.0 (5.11)	18. 5 (3.3)	18.4 (5.1)
12 month Nonverbal Standard Score	57.0 (6.3)	55.6 (5.4)	46.8 (9.3)*
36 month Verbal Standard Score	58.1 (5.6)	54.1 (7.0)	48.6 (10.7)*
36 month Nonverbal Standard Score	60.0 (8.0)	60.3 (8.2)	47.1 (14.4)*
ADOS social total	N/A	3.1 (1.7)	8.7 (5.9)
ADOS communication total	N/A	2.6 (1.8)	8.5 (4.3)
ADI-R Social	N/A	3.1 (.5)	6.2 (1.3)
ADI-R Communication	N/A	2.6 (.6)	7.3 (1.2)
ADI-R SBRI	N/A	.6 (.3)	3.7 (.5)

Table S4: Profile of infants included in analyses. Values are mean (standard error).

3.3.1 Results Trial Number: A chi-squared analysis showed no group differences in the proportion of children who had data from one or both videos (x^2 = 2.24, p = 0.33). In a repeated measures ANOVA on the number of segments included in the first and second halves of the videos, trial yields from the first half were significantly greater than for the

second half ($F(1,34) = 10.6$, $p = 0.003$, $\eta^2 = 0.24$). This did not interact with group ($F(2,34) = 0.54$, $p = 0.6$), and there was no overall group difference in trial numbers ($F(2,34) = 0.54$, $p = 0.59$).

Analyses were repeated, covarying for the number of trials derived from the first and second halves of the video. The pattern of results was the same. Specifically, greater change in frontal theta related to higher nonverbal cognitive skills at 36 months across groups ($F(1,34) = 10.97$, $p = 0.003$, $\eta^2 = 0.30$). There was also a significant interaction with group ($F(2,34) = 4.77$, $p = 0.017$, $\eta^2 = 0.27$). Greater change in frontal theta also related to higher verbal skills, but this varied by group ($F(1,34) = 3.53$, $p = 0.044$, $\eta^2 = 0.21$); overall effect ($F(1,34) = 2.44$, $p = 0.13$, $\eta^2 = 0.09$).

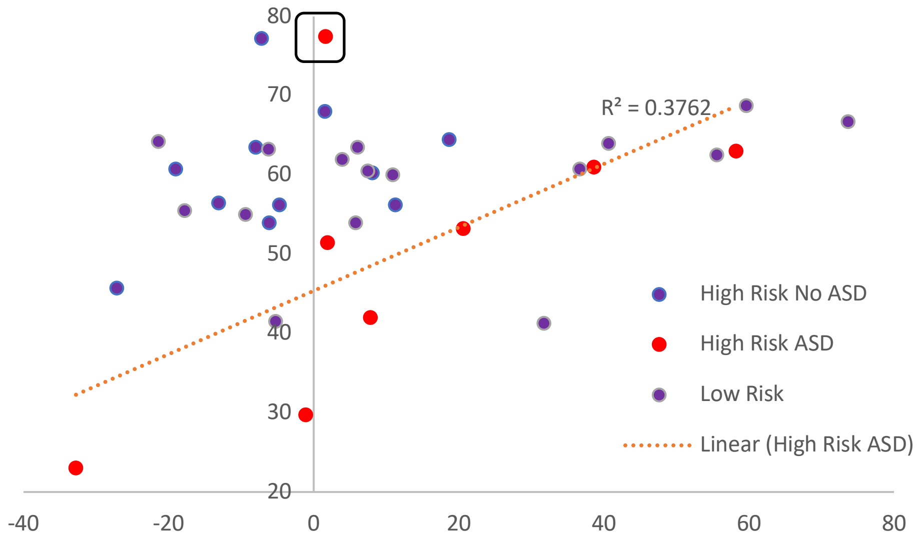

Supplementary Figure 1: Bivariate outlier

3.3.2 Results Bivariate outlier: Figure S1 illustrates the bivariate outlier for theta change and nonverbal skills (circled). This child was considered a "borderline" case in the 3-year diagnostic assessment and did not receive a diagnosis of autism in a clinical setting; the child did not return at age 7 years and hence stability of his profile cannot be ascertained.

Analysis including this child shows that greater change in frontal theta related to higher nonverbal cognitive skills at 36 months across groups ($F(1,35) = 7.26$, $p = 0.012$, $\eta^2 = 0.2$, controlling for 12-month nonverbal skills $F(1,34) = 3.42$, $p = 0.048$, $\eta^2 = 0.21$). The interaction with group becomes nonsignificant ($F(2,35) = 2.39$, $p = 0.1$, $\eta^2 = 0.14$).

4. Cohort 3, 7-year follow-up

4.1 Further participant information: Of 53 HR and 48 LR children retained at the 3-year assessment, 44 HR (83%) and 37 LR (77%) agreed to take part in the follow-up study. Of these, two HR children did not complete a research visit (parents completed questionnaires only). As we did not see these children we were unable to assign them to an ASD outcome group and consequently excluded them from the current analyses, leaving a final sample of 42 HR siblings (15 boys, 27 girls) and 37 LR controls (14 boys, 23 girls). The HR and LR groups did not differ in age (HR mean (SD): 90.6 (6.3) months; LR mean (SD): 89.3 (4.9) months; $t(74) = -1.00$, $p = .31$) or sex (HR % male: 35.7; LR % male: 37.8; $\chi^2(1) = .038$, $p = .85$) at the follow-up. The retained sample did not differ from the non-retained sample in 3 year levels of ASD on the ADOS, SRS, or SCQ, developmental level on the MSEL, adaptive behaviour assessed with the Vineland Adaptive Behaviour Scales – Second Edition (Vineland-II; Sparrow et al., 2005), or family income (all $p > .4$). Parents provided written informed consent. Children provided written informed assent wherever possible given developmental level.

4.2.1 Methods- Measures: The Wechsler Abbreviated Scale of Intelligence – Second Edition (*WASI-II*; Wechsler, 2011), a standardised instrument to assess intellectual ability, was completed with each child. Standardised, age-normed intelligence quotients (mean 100; SD 15) for the verbal domain (Verbal Comprehension Index, VCI), performance domain (Perceptual Reasoning Index, PRI), and full-scale IQ (FSIQ) were used in analyses. One HR child was unable to complete the assessment due to intellectual disability.

4.2.2 Methods- Autism Assessment: Experienced researchers who conducted the assessments (ES, BM, GP) and the lead clinician (TC) reviewed information on ASD symptomatology (ADOS-2, ADI-R (HR only), SCQ) and adaptive functioning (Vineland-II) for each HR and LR child and as a team assigned clinical consensus best estimate diagnosis of ASD according to DSM-5 (American Psychiatric Association, 2013). None of the LR children met DSM-5 criteria for ASD and none had a community clinical ASD diagnosis. Diagnosis at age 7 years included review of all information previously obtained and there was overlap in the personnel involved in the diagnostic decision-making (GP, TC). However, the age 7 diagnostic decisions were not directly yoked to the diagnostic decisions previously taken at the 3-year visit but rather, in light of the large amount of additional information available about the children – in particular with respect to peer interactions and functioning outside the home setting – an independent decision was made as to whether the child currently met DSM-5 criteria for ASD.

4.2.3 Methods - Final sample: Of the original sample of 16 low risk infants and 18 high risk infants with EEG data at 12 months, 11 low risk and 14 high risk infants had data available on the WASI at age 7 years. Of note, we used risk group rather than outcome group in this analysis because of the small sample size and because some children changed diagnostic category between age 3 and age 7[6]. Specifically, of the 14 children in the high-risk group four were considered to have ASD at both 3 and 7, one at 3 not 7, and one at 7 not 3. In Figure 3E children with an ASD diagnosis at 7 (concurrent with the IQ data) are shown in red; children who changed diagnostic status are indicated with arrows.

1. Jones, E. J. H., Venema, K., Lowy, R., Earl, R. K. & Webb, S. J. Developmental changes in infant brain activity during naturalistic social experiences. *Dev. Psychobiol.* **57**, 842–853 (2015).
2. Jones, E. J. H. *et al.* Reduced engagement with social stimuli in 6-month-old infants with later autism spectrum disorder: a longitudinal prospective study of infants at high familial risk. *J. Neurodev. Disord.* **8**, 7 (2016).
3. Jones, E. J. H., Dawson, G., Kelly, J., Estes, A. & Webb, S. J. Parent-delivered early intervention in infants at risk for ASD: Effects on electrophysiological and habituation measures of social attention. *Autism Res.* **10**, 961–972 (2017).
4. Orekhova, E. V. *et al.* EEG hyper-connectivity in high-risk infants is associated with later autism. *J. Neurodev. Disord.* **6**, 40 (2014).
5. Haartsen, R., Jones, E. J. H., Orekhova, E. V., Charman, T. & Johnson, M. H. Functional EEG connectivity in infants associates with later restricted and repetitive behaviours in autism; a replication study. *Transl. Psychiatry* **9**, 66 (2019).
6. Shephard, E. *et al.* Mid-childhood outcomes of infant siblings at familial high-risk of autism spectrum disorder. *Autism Res.* **10**, 546–557 (2017).
